# Going It

**Published:** 1882-01

**Authors:** 


**"GOING IT."**—The perfectly reckless manner in which human health and hopes and life itself are trifled with, finds a powerful illustration in the narration of a banker, given with all the directness, succinctness, and matter-of-fact way of a thoroughly business man :

"It was my habit to smoke what was equivalent to a dozen segars a day. I also drank a good deal, and for the five years preceding my sickness, I had been much confined to business, scarcely having any exercise, also slept at place of business, where the air was very bad. Add to these the fact that I did not know any thing about taking care of myself."

It was with the hope that parents would be glad to supply their children with a JOURNAL OF HEALTH like this, in order to give them in time the very information which would have saved the banker long years of illness, with all the sufferings, anxieties, and vain labors for health which have followed in the train. And yet it is very certain, that not one parent in any hundred on an average, who reads this article, will spend a dollar for this or any similar publication for a son at college, or a daughter at a boarding-school, or will devote one hour in a month towards instructing their children at home, as to the means of maintaining health or of avoiding disease. And yet, when health is lost, these very same parents, looking on the flower early and surely fading away into the grave, will freely spend hundreds and thousands of dollars, will eagerly undertake painful journeys by land, and encounter the perils of ocean navigation for thousands and thousands of miles, for the bare chance of a little improvement, and the phantom-like hope of an eventual restoration. It certainly is not desirable to fill the minds of the young with symptoms and remedies. Such is not the object of this or publications like it, but to show, in every variety of way, how diseases are engendered, and how, by a trifling care and a little wise attention, to avoid them. May the day soon come when it will be considered a better "start in the world," to enter upon the great theater of human life at "twenty-one," with a good constitution in a high state of preservation, than to "begin business" with a large capital and no "physique," no bodily vigor to carry it on with energy, activity and life.

